# Mathematical modeling of movement on fitness landscapes

**DOI:** 10.1186/s12918-019-0704-0

**Published:** 2019-02-28

**Authors:** Nishant Gerald, Dibyendu Dutta, R. G. Brajesh, Supreet Saini

**Affiliations:** Department of Chemical Engineering, Indian Institute of Technology Bombay, Powai, Mumbai, 400 076 India

**Keywords:** Fitness landscape, Optimal parameters, Cost-benefit framework, Gene regulatory network

## Abstract

**Background:**

Movement of populations on fitness landscapes has been a problem of interest for a long time. While the subject has been extensively developed theoretically, reconciliation of the theoretical work with recent experimental data has not yet happened. In this work, we develop a computational framework and study evolution of the simplest transcription network between a single regulator, R and a single target protein, T.

**Results:**

Through our simulations, we track evolution of this transcription network and comment on its dynamics and statistics of this movement. Significantly, we report that there exists a critical parameter which controls the ability of a network to reach the global fitness peak on the landscape. This parameter is the fraction of all permissible values of a biochemical parameter that can be accessed from its current value via a single mutation.

**Conclusions:**

Overall, through this work, we aim to present a general framework for analysis of movement of populations (and particularly regulatory networks) on landscapes.

**Electronic supplementary material:**

The online version of this article (10.1186/s12918-019-0704-0) contains supplementary material, which is available to authorized users.

## Background

Movement of populations on fitness landscapes has been a topic of interest for a long time. Since first proposed by Wright [1], fitness landscapes have offered a tool for visualization of how populations enhance their fitness with time, and move towards local/global peaks [2, 3]. However, despite a large volume of theoretical development of representations of landscapes, few realistic representations exist [4–8]. This is most strongly due to the challenges associated with gathering enough experimental data to build an appropriate landscape [9–13]. A few, recent efforts in this direction have highlighted the challenge in building experimental systems to provide sufficient information for our understanding of fitness landscapes of real systems [14] .

While the experimental treatment of this subject is still small, and theoretical contributions becoming increasingly rare; alternate approaches to visualization can be of assistance in understanding landscapes and movement of populations on them. In this regard, while, given a fitness landscape, the rules which dictate a population’s movement on that landscape are well known; the primary challenge stems from limited understanding of the precise structure on which populations are supposed to be moving. In this regard, in a report published in *Nature*, Draghi and colleagues developed a quantitative framework for understanding the relationship between the variables robustness and evolvability [15]. Previous work from our group quantified how, using a cost-benefit framework, organisms choose and optimize the value of parameters in biochemical networks [16].

**Fig. 1:**
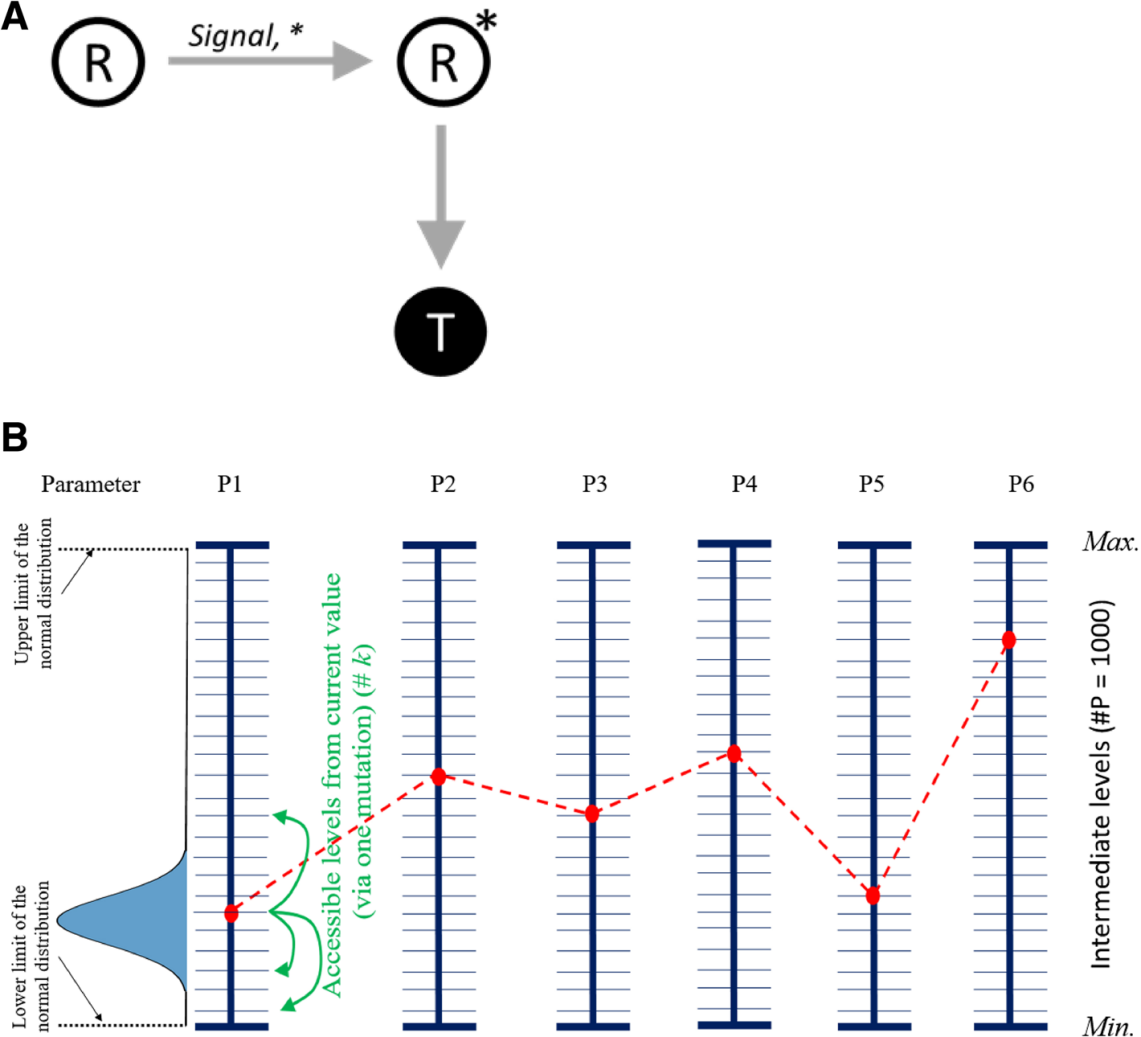
**a** The regulatory network simulated in this work. The protein R, in the presence of signals, acts as a transcription factor, R*, and activates expression of the target protein, T. **b** The simulation scheme. Each of the six parameters was allotted a distinct minimum and maximum value, and 998 other distinct values in the range. The red dots represent one such parameter set. For each parameter, from its current value, movement via a single mutation is only permitted to k distinct values (highlighted in green). The likelihood of a value being taken by the parameter, post mutation, is given by a normal distribution centered around the current value of that parameter. In this work, P1 is bas (eq. 1); P2 is k (eq. 1); P3 is kd (eq. 1); P4 is β (eq. 3); P5 is K_m_ (eq. 3); and P6 is k_dT_ (eq. 3). To reduce the computational complexity of the set-up, in this work, k_dR_ was taken to be 0.001 times maximum value of k_d_ in all parameter sets

Borrowing from these two reports, we develop a quantitative framework to analyze movement of populations on fitness landscapes, and more importantly, how is this movement dictated by the precise nature of the landscape. We, as reported earlier, use the simplest transcription factor network possible (between a single regulator *R* and a target protein, *T*) (Fig. 1a) [16]. Proteins *R* and *T* serve a physiological function in the cell, and hence we link their expression levels with the benefit conferred to the cell. However, protein expression in a cell is costly, and maintaining *R* and *T* incurs costs too. We develop expressions for these cost and benefit and link them with the fitness contribution towards the cell.

In our study, we focus particularly on two variables associated with the landscape and the population. First, the connectivity of the landscape, *k*. By this, we mean the fraction of fitness levels (among all) that an organism can access via acquisition of a single mutation. The second variable is the fitness associated with the population at that instant, *f*_*0*_ (see methods for more details).

In particular, in this work, we focus on the impact of the structure of the landscape on the movement, and do not take into account effects of stochasticity (such as drift), which allow populations to move through valleys on landscapes. Through our work, we show that there exists a critical value of the connectivity parameter, *k*, beyond which populations are almost certain to reach the global peak in the landscape. Below this critical value of *k*, the populations are almost always likely to get “trapped” in local optima. Such a sharp transition in the probability to reach the global peak with changing *k* represents an inherent property of the graph associated with the fitness landscape. In addition, we also comment on the time to reach fitness peak and the predictability associated with a population’s movement on the landscape.

## Results

### Evolutionary trajectories reach the global peak on the fitness landscape

**Fig. 2:**
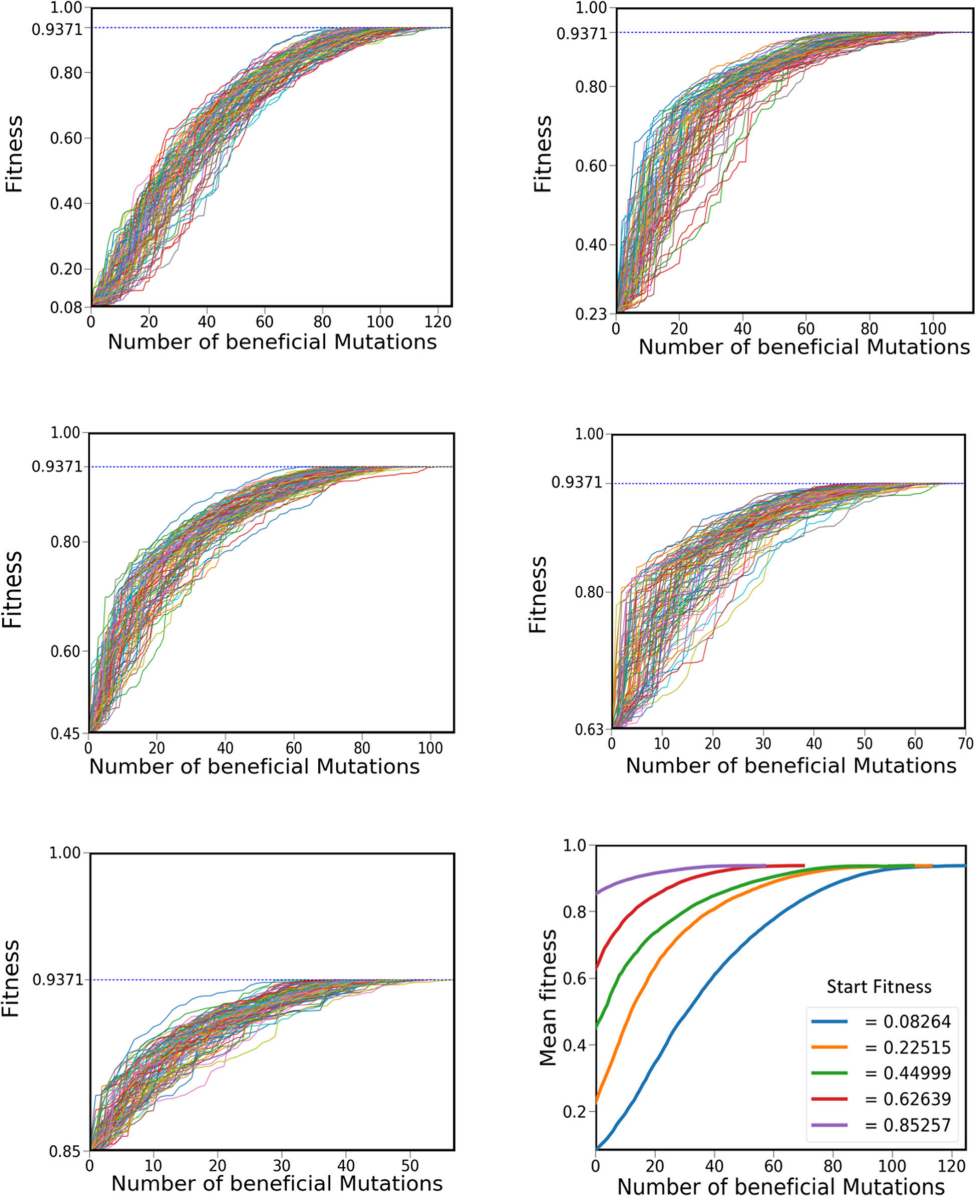
The dynamics of increase in fitness of the R-T transcription network. Dynamics of 100 different trajectories when starting from a parameter set which corresponds to a unique initial fitness. (Top left) Starting fitness of 0.08264. (Top right) With starting fitness of 0.22515. (Middle left) For starting fitness 0.44999. (Middle right) For starting fitness 0.62639. (Bottom left) For starting fitness 0.85257. (Bottom right) Average of the hundred trajectories for each of the five parameter sets

Our model is a six dimensional parameter space, where each is allowed to change according to set rules (see methods section). These rules defining how changes in parameter values are acquired, we feel, represent the biochemistry of alterations in promoter/protein function. In the first set of simulations, we set the value of parameter *k* = 50; that is, each parameter is allowed to move to, on an average, 50 different values (out of a total of 1000). Later in this work, we report how changing the value of *k* changes the results from these simulations. For this first simulation, we choose a parameter set with a corresponding fitness of 0.08. This value represents about 12% of the maximum possible fitness (0.9371) on our fitness scale. From this starting parameter set, we let the system evolve by changing one of the parameter values at a time, thereby, leading to movement of the population towards higher fitness values. As shown in Fig. 2a, we note that after a large number of mutations in the simulation, the system is able to reach the global fitness in the scale used in this study. We repeat this process a hundred times and the resulting trajectories from each simulation are as shown in Fig. 2.

**Fig. 3:**
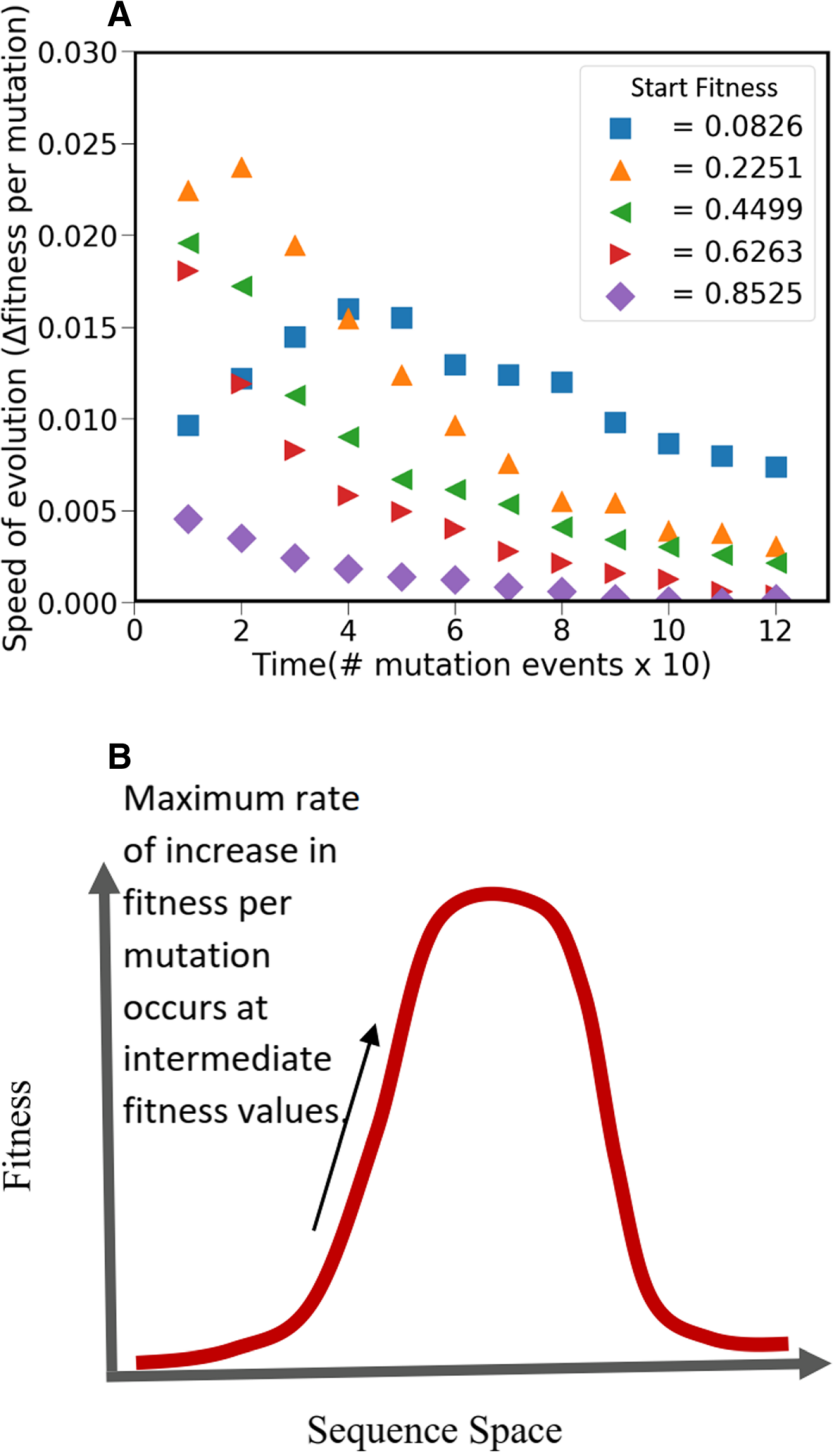
(**a**) Speed of evolution (increase in fitness) with increasing fitness of the parameter set. For all initial starting fitness, the rate of increase in fitness decreases as time (or the current fitness) increases. However, for small initial fitness (0.0826), the first phase of the evolutionary trajectory includes potentiating mutations which facilitate a more rapid increase in fitness. This phase is followed by the second phase where the rate of increase of fitness decreases with time. (**b**) Speed of evolution is maximum at intermediate fitness values. The trajectory shown by small initial fitness (blue filled square in Fig. 3(**a**)) can be visualized as movement on a landscape whose shape is characterized by the peak as shown in the lower panel. In such a scenario, the speed of evolution (fitness gain per mutation) is maximal at intermediate fitness values, and is lower for both cases, when fitness is too high or too low

Next, we repeat this procedure for five distinct values of starting fitness, and note a similar trend (Fig. 2a-e). The mean fitness of the hundred trajectories are as shown in Fig. 2f. From this data, we calculate the speed of evolution (rate of increase of fitness), and note that the maximum rate of increase of fitness is observed when the starting fitness of the set is intermediate (0.23) (Fig. 3). We speculate that this happens because when starting from low values of fitness, the first few mutations are potentiating mutations [17]. These mutations do not themselves increase the fitness of the system qualitatively, however, they provide the parameter set to thereafter acquire new beneficial mutations and undergo maximal rate in change in mean fitness. We also note that, for *k* = 50, with increasing initial fitness, there is a statistically significant decrease in the number of steps needed to reach fitness peak on the landscape. Perhaps another way to think about this result is to imagine the fitness peak in the landscape associated with the network as a normal distribution (see Fig. 3). In such a setting, the first few beneficial mutations confer a lower fitness advantage, compared to the intermediate mutations.

On the whole, our results suggest that in a fitness landscape of rather small dimensionality, there exist a large number of paths to reach the global fitness peak. Each trajectory explored in our study is unique in its fitness levels, and the order of mutations it acquires.

### The value of *k* indicates a critical transition in the network’s ability to reach the fitness peak

Next, we were interested in studying how changing the value of *k* changes the dynamics of this process. To explore this, we repeated the simulations described in the previous section for *k* = 3, 5, 7, 10, 12, and 20. Our naïve assumption before performing these simulations was that as the value of *k* decreases, the fraction of times the fitness is able to reach the peak on the landscape would reduce linearly. Increasingly, with decreasing *k*, the population would get trapped at a local peak. However, prior to performing the simulations, we could not comment on the precise nature of this decrease observed with increasing *k*.

**Fig. 4:**
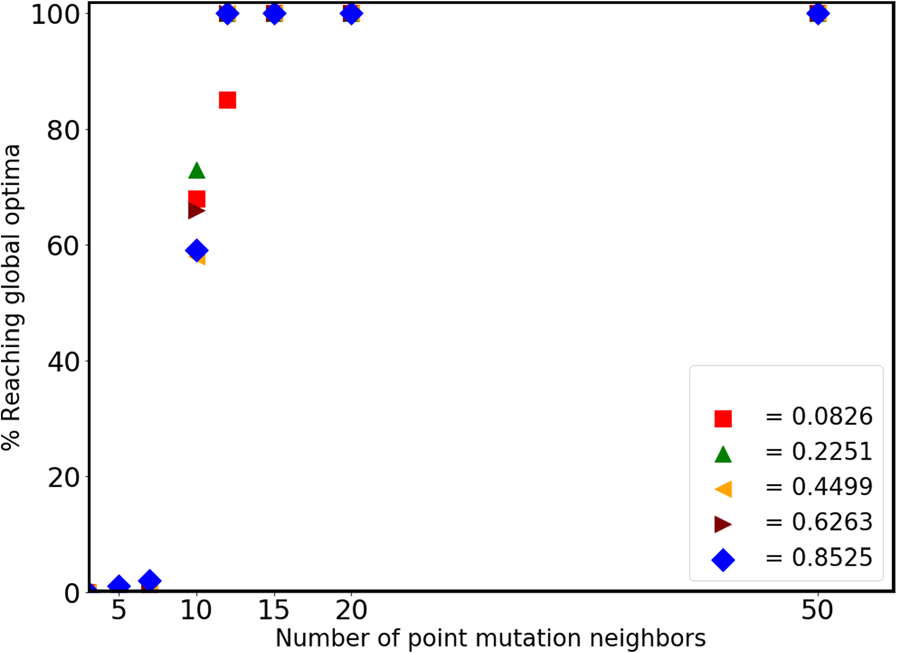
The likelihood of reaching the peak fitness increases from zero to one around a critical value of k. Independent of the starting fitness corresponding to a parameter set, the likelihood of reaching the global fitness peak on the landscape increases from zero to one as the value of k increases beyond 10. The y-axis represents the average of 100 distinct simulations for each set of parameter values and a k value

As shown in Fig. 4, with increase in the value of *k,* there is a sharp transition in the fraction of trajectories that are able to reach the maximal fitness on our landscape. Our data shows that at low values of *k*, the network dynamics are such that the system is almost always never able to reach the fitness peak. However, as the value of *k* increases (beyond 10), the system is almost always able to reach the peak fitness. Since 1000 levels exist for value of each parameter, this suggests that an access for 1% of all fitness levels is able to ensure that there is a Darwinian path to maximal fitness.

Interestingly, the transition from a zero probability of reaching the peak fitness (at *k <* 10) to a probability of one (at *k >* 10) becomes sharper as the fitness of the starting parameter set decreases. This is likely because at high starting fitness, if there does not exist a direct connection to a higher fitness; then the system will likely be stuck at that fitness level. At lower starting fitness, however, the chances of there being access to higher fitness will be high – leading to eventual access to the peak fitness. To test the possibility whether this result was dependent on the exact fitness function chosen, we performed a number of simulations with altered values of *a* and *b* in the fitness function. As shown in Additional file 1: Figure S1, value of *k* at which the population trajectories reach the global peak is invariant with respect to the fitness function. Thus, our results show that this property is an inherent property of the graph being analyzed.

### Time to reach peak fitness indicates a critical transition

Although all values of *k* greater than 10 are able to reach the fitness peak, the time to do so varies significantly. In general, the greater the value of *k*, the lesser the time (in terms of number of mutational events) to reach the fitness peak. However, as in the previous section, in this result too there is a critical value of *k* beyond which the time to reach the fitness peak changes qualitatively.

**Fig. 5:**
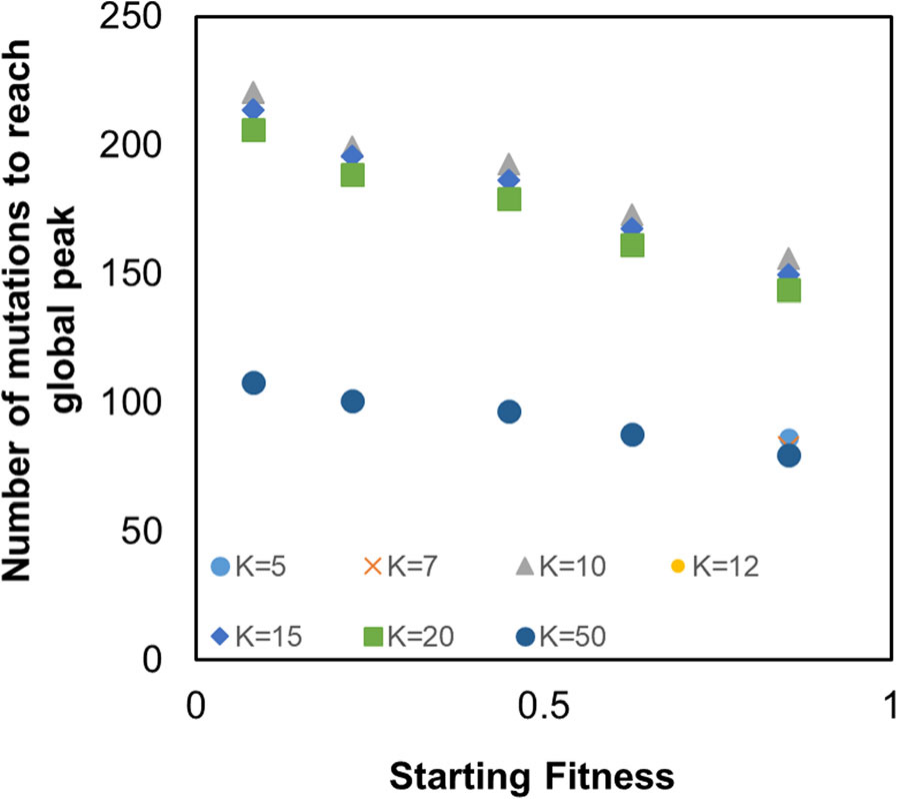
The mean number of mutational events needed to reach the global fitness peak decreases linearly with increasing starting fitness of the parameter set. The data is average of 100 distinct simulations for each set of parameter values and a k value. For K equal to 5 and 7, for all starting fitnesses (except 0.8525), the populations get trapped in local optima and never reach the global peak. As a result, the number of mutations to reach global peak is infinite, and hence, not shown on the graph

As shown in Fig. 5, we plot the number of mutational events needed (on an average) to reach the fitness peak for different values of *k* and different starting fitness. In general, the number of steps needed to reach the fitness peak decreases linearly as starting fitness increases. However, the time taken reduces qualitatively beyond a specific value of *k*. For small values of *k* (< 10)*,* the number of steps needed to reach the global fitness peak is infinite. Moreover, beyond that, our data shows that as the value of *k* changes from 10 to 20, the time to reach the fitness peak changes (reduces) by less than 10%. However, as we next change the value of *k* to 50, the value of time taken to reach the fitness peak changes (decreases) by almost 100%. This qualitative change in behavior, interestingly, occurs at a value of *k* which is different from the transition value of *k* from the previous section (where the percent of times the system is able to reach the fitness peak changes from 0 to 100%).

### Predictability or randomness in the evolutionary trajectories

One of the questions we were interested in addressing through our framework was that of predictability of the trajectories that starting parameter sets follow. In this regard, we set up simulations for a starting point of the parameter set, **P** and the associated value *k.* The simulation from this starting point was run a 100 times and the difference in dynamics recorded. Prior to analysis of our trajectories, we anticipated the following result: starting points where trajectories move towards both, local and global optima will show a non-zero variance in the values of fitness at steady state (where all 100 trajectories have reached an optima). On the other hand, starting points which enable all trajectories to reach the global peak will lead to zero variance at the time when all trajectories have reached fitness peaks.

**Fig. 6:**
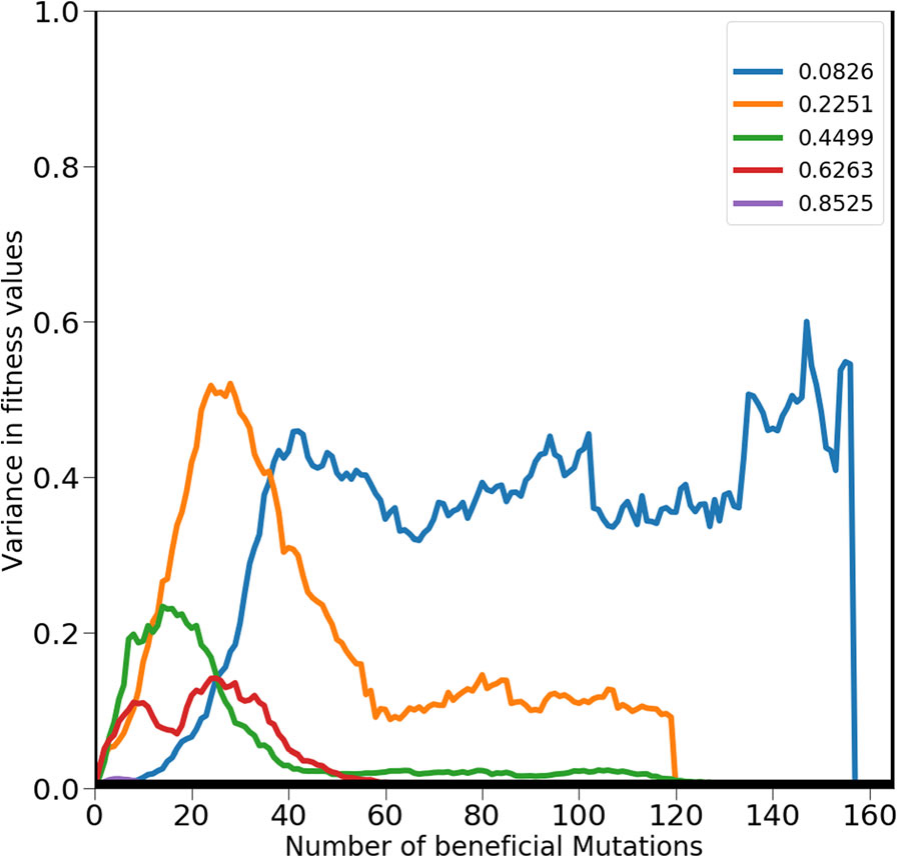
The dynamics of variance in fitness between the 100 trajectories for each parameter set a k value. For the smallest starting fitness value (0.0826), the peak variance is achieved much later as compared to the other starting fitness. This is due to the first phase of the fitness trajectory includes potentiating mutations. The data shown is for a *k* value of 15. Other values of k show qualitatively similar trajectories (data not shown)

As shown in Fig. 6, the above mentioned intuition is reflected in our results. For the starting point which corresponds to all trajectories reaching the global peak, we note an increase in variance of fitness values between trajectories, as the trajectories diverge from the starting point (**P**, *k*). Thereafter, as all trajectories converge towards the global peak, the variance converges to zero. This result is analogous to the classical fitness experiments from Lenski’s LTEE experiments from the early 1990s [18]. On the other hand, the starting points which do not lead all trajectories to the global optima result in a non-zero, finite non-zero variance is observed at the end of the experiment (when all trajectories have reached fitness peaks).

Interestingly, the variance among trajectories which start from a lower fitness is qualitatively higher than the variance between trajectories which start from a higher value. This result is consistent among all values of *k* tested in this work. Intuitively, this is likely because starting at a lower value of fitness, the parameter set has an exponentially greater number of trajectories to follow from (compared to another starting point at a higher fitness). As the fitness of the starting set increases, the number of options available to acquire a mutation that leads to an increase in fitness decrease. Hence, the variance is much higher among trajectories starting from a lower fitness, as compared to those starting from higher fitness values. This result is perhaps best understood from the mountain climbing analogy. At the foot of the mountain, the number of paths leading to the top are very many. However, close to the top, there are only going to be a few (or one) paths leading to the summit.

Secondly, as discussed in the previous section, the first few mutations to the parameter set which corresponds to the lowest fitness are “potentiating mutations”. These mutations, as discussed above, do not lead to a great increase in fitness but prepare the set for acquisition of mutations which lead to a much greater increase in fitness. As a result, although the greatest variance is seen in starting points where **P** corresponds to lowest fitness; the variance among the trajectories starting from **P** increases after a brief lag. This lag corresponds to the period where the “potentiating mutations” are being acquired by the set.

## Methods

### Model system

The model used in this work is the simplest transcription factor network in bacteria – a single regulator, *R* and a single target protein, *T* (Fig. 1a). In presence of appropriate environmental signal, *s*, the transcription factor gets transformed to its active state, R*. In its active form, R*, working as a dimer, is able to control expression of the target protein, *T.* The dynamics of this process can be represented by the following equations.

1 $$ \frac{dR}{dt}= bas-k.R.S+{k}_r.{R}^{\ast }-{k}_d.R $$

2 $$ \frac{d{R}^{\ast }}{\mathrm{d}t}=k.R.S-{k}_r.{R}^{\ast }-{k}_d.{R}^{\ast } $$

3 $$ \frac{dT}{dt}=\frac{\beta .{R}^{\ast 2}}{K_m^2+{R^{\ast}}^2}-{k}_{dT}.T $$

where, k is the rate of conversion of *R* to R^*^; k_r_ represents the rate of conversion of R^*^ to *R*; k_d_ is the rate of degradation of the regulator *R*; β is the maximal rate of expression of the target protein *T* (when supply of *R* is infinite); k_m_ corresponds to the regulator concentration at which target protein is expressed at half its maximal rate; and *bas* is the basal expression level of the regulator. This dynamic representation of the model assumes that the regulator expression is not regulated. On the other hand, the target production is controlled by the regulator. More precisely, in the presence of the signal, the regulator molecule interacts with the cue and changes to its functional form R*. The active form then forms a dimer and interacts with the operator site in the promoter of target gene, leading to expression of the parget. Both regulator and the target are assumed to degrade and diluted because of growth, and this process is quantitatively captured as first order kinetics.

The benefit and cost associated with expression of *R* and *T* can be represented as the following. While alternate qualitative expressions of benefit curves are known to exist [16], in this work, we work with the most intuitive representation of the benefit curve associated with a target protein production in a cell. When the production of target starts in the cell, the rate of increase in the benefit that the cell derives is maximal. However, with increasing production of the target protein, the incremental benefit for the cell decreases. This diminishing return of benefit with increase target amounts is captured by the following expression. We note, however, that there could also be scenarios where the target acts on physiology as a dimer, or that excessive production of target is detrimental to the cell (e.g., via accumulation of a toxic metabolic intermediate). In either of these two settings, the benefit function represented in Eq. (4) will not be representative. However, the expression below captures the physiology of most proteins in bacterial physiology.

4 $$ B=\frac{a\ast T}{T+b} $$

This benefit function (*B*), with increasing target protein concentration, is assumed to be an increasing and a saturating function. The constant *a* was assumed to be 1, and *b* as 10. Other values of the variables were also taken. The results for those simulations are as shown in Additional file 1: Figure S1.

5 $$ C=\propto \ast \left(T+{R}^{\ast }+R\right) $$

Where, α represents the cost per molecule times the degradation constant of a protein. Collectively, this ensures that the expression for cost is the number of protein molecules needed to be synthesized per unit time to maintain steady state levels of *R* and *T* times the cost incurred per protein molecule. The value of α was taken as 2.5 × 10^− 5^.

From these two expressions, the fitness of the individual, *F*, was defined as the difference between the benefit and cost values, as shown below.

6 $$ F=B-C $$

Since in the fitness calculations we are only making use of the steady state expression values of R and T, we note that our analysis is valid for situations where the signal s is time invariant. For example, enteric bacteria, once they enter the body, face a more or less constant temperature and oxygen concentration.

### Neighbor-network

To define the fitness landscape associated with the parameter values, the following approach was used. Parameters were allowed discrete values, the range of each value was confined to a *min* and a *max* value, which are based on the thermodynamics of the biological processes represented by each parameter (Additional file 1: Table S1) [19–21]. Between the *min* and *max* value for each parameter, 998 unique values were chosen randomly. These 1000 values collectively represented the set of values that a particular parameter is allowed to take (see Fig. 1). The range of the values permissible for the parameters were taken from previous works which have identified physiologically relevant constraints on these biochemical parameters [19–25].

However, from its current value, a parameter was not permitted to take any of the remaining 999 values via acquisition of a single mutation. Instead, we define a variable called connectivity, *k* which defines the number of discrete values that the parameter can acquire post a single mutation. For example, if the value of *k* is 50, the parameter can go to 50 distinct values of *k* after acquiring a mutation. The remaining 949 levels remain inaccessible to the parameter, via a single mutation.

After defining the value of *k*, we next devised a strategy to decide which 50 of the 999 values are accessible to a parameter via a single mutation. To do this, a Gaussian distribution, centered on the current value of the parameter and with a sigma value of 2% of the 1000 (i.e. 20), was defined. This Gaussian distribution defined the probability of a particular value of a parameter being accessed from its current value of that parameter in a single mutation. Using this distribution, thereafter, 50 distinct values of the parameter were chosen as a one-mutation distance neighbors of the current level. By definition, if value *i* was neighbor of value *j;* automatically, *j* was allotted to be a neighbor of value *i.*

### Locating global or local optimal positions for parameter vectors

Based on the model formulation, there are seven parameters in our model. To reduce the dimensionality of the problem, we assume that *kr* is equal to 0.001 times *k* [16]*.* The other six parameters are allowed to take on values in the range as given in Additional file 1: Table S1.

Each of the six parameters was permitted to take 1000 values between its minimum and maximum permissible values. Previous work in this direction has treated parameter values as a continuous variable. In this work, however, we argue that discrete values are more representative of physiology of biochemical parameters, and hence, only permit discrete values of parameters. As a result, the parameter set (consisting of six parameters) could take one of 1000^6^ values.

From a particular value of a single parameter, it was allowed to move to any one of the *k* pre-determined values, by acquisition of a single mutation. Which means that a particular parameter set had 6 *k* one-mutant neighbors associated with it. Each of these mutant sets corresponds to a particular value of fitness. To locate the trajectory and the fitness optima associated with a particular set, the following was done.

Starting from the original parameter set, all 6 *k* one-mutant neighbors were analyzed for their fitness. Thereafter, the resulting mutations (and mutants) were characterized as beneficial or deleterious (depending on their impact on the fitness). Of all the beneficial mutations, one was chosen randomly (with uniform probability), and the parameter set assumed to move to the value associated with this beneficial mutation. This process was then repeated for the new parameter set. Once a parameter set was reached such that all 6 *k* neighbors were of fitness lower than the root set, it implied that the particular trajectory had reached a global or a local maximum of fitness. The corresponding parameter values, the value of the fitness were, thereafter, recorded.

We note that we do not take into consideration effects like drift, and the consequent fact that mutations with stronger benefit have a higher likelihood of surviving drift. We also ignore in this framework that occasionally, even deleterious mutations could establish themselves and thereafter, go to fixation; particularly if the population size is small. In this work, we do not take into account these two factors, as we only address the question of the likelihood of reaching a fitness peak in a selection-dominated framework, and ask how does the network structure impact this movement?

### Simulation scheme

In this work, the dynamic trajectories were computed for the following conditions. Eight different values of the variable *k* were chosen: 3, 5, 7, 10, 12, 15, 20, and 50. Parameter sets corresponding to five different initial fitness were chosen. The fitness values were: 0.0826, 0.2251, 0.45, 0.6264, and 0.8526. Our premise behind these choices was to cover dynamics of trajectories starting from highly diverse starting fitness values, varying from very low (0.0826) to very high (0.8526). In our system, the global optimum has a fitness of 0.9371.

Finally, to track the diversity of trajectories starting from the same location in the parameter space, dynamics of population movement from each starting point (defined by the parameter set **P** and *k*) was tracked a 100 times. The trajectories associated with each were noted, and are as presented in the results section. All simulations were performed on Python version 3.6.

## Discussion

In this work, we develop a framework to answer the following question: how does the network connectivity (the fraction of nodes one particular node is connected to) influence the ability of a network (or an organism) to reach the peak fitness on a landscape? The question is relevant since biological parameters, since they are sequence dependent, are discrete variables, and from a given position, can only move to a fraction of all permissible values. We develop the framework to answer this question and note that there is a sharp transition in the network’s ability to reach the global peak at a particular value of the connectivity variable, *k.* From our work, we note that at a value where a node is able to access 1% of all nodes via a single mutation, the network is able to access the global peak almost 100% of the times. Below the 1% connection, the network is almost never able to reach the global peak, and evolutionary trajectories get trapped in a local optima. These results suggest a link between the connectivity, *k* and the dimensionality of the network (in this case six). We anticipate that for networks with higher dimensionality, the critical value of the connectivity parameter would be less than 1%. This is likely to be so because higher dimensions would offer qualitatively different number of routes for populations to not get “trapped” in local optima.

Since the number of values a biological parameter can take is constrained by thermodynamics and biochemistry, it is likely to be independent of the network. This implies that larger networks (with a greater number of parameters) would have a greater likelihood of reaching global peak as compared to smaller networks (with smaller number of degrees of freedom). To test this, we performed a similar set of simulations with the lactose utilization network in *E. coli*, and show that for this network, where the number of degrees of freedom is 10, exhibits the critical transition (from a near zero probability of reaching the global peak to a near one probability of reaching the global peak) at a lower value of *k* (at 0.25%) (*data not shown*). Thus, higher connectivity between networks is likely to result in a greater likelihood of reaching a global peak on fitness landscapes.

One of the crucial assumptions in this work is the fact that the likelihood of the new value of a parameter, post mutation, is distributed normally centered around the current value. The distribution of mutations has been a topic of interest of a number of experimental and theoretical studies, but remains an open question [26–31]. In a recent work from our group, we have developed a computational framework for analysis for studying these distributions (*in review*). Our results suggest that exponential or normal distributions can statistically approximate the distribution of mutational effects to a satisfactory degree. This is especially true when the starting fitness corresponding to a particular set is low (compared to the peak permissible fitness). What distribution of a parameter value in its prescribed range results in an exponential or a normal distribution, however, remains an open question.

## Additional file

Additional file 1: **Figure S1.** The likelihood of reaching global optima increases from zero to one around critical value of K when the benefit is higher than the cost. In this simulation the parameter (A) *a* is increased by a factor of 10; (B) parameter associated with cost per protein molecules (*α*) is increased by a factor of 10; (C) the parameter associated the sensitivity of the benefit function (*b*) is increased by a factor of 10; and (D) the parameter associated the sensitivity of the benefit function (*b*) is decreased by a factor of 10. **Table S1.** Parameter range of all 6 parameters (k, k_r_, k_d_, b, k_m_, bas) used in the simulations. (DOCX 503 kb)
