# Supplementary material for: Mathematical modeling of movement on fitness landscapes
Source: BMC Syst Biol. 2019 Feb 28;13:25. doi: 10.1186/s12918-019-0704-0 (PMC6394095; doi:10.1186/s12918-019-0704-0)
Supplement: Supplementary file 1 — Figure S1. The likelihood of reaching global optima increases from zero to one around critical value of K when the benefit is higher than the cost. In this simulation the parameter (A) a is increased by a factor of 10; (B) parameter associated with cost per protein molecules (α) is increased by a factor of 10; (C) the parameter associated the sensitivity of the benefit function (b) is increased by a factor of 10; and (D) the parameter associated the sensitivity of the benefit function (b) is decreased by a factor of 10. Table S1. Parameter range of all 6 parameters (k, kr, kd, b, km, bas) used in the simulations. (DOCX 503 kb) [file 12918_2019_704_MOESM1_ESM.docx]

**SUPPLEMENT: MATHEMATICAL MODELING OF MOVEMENT ON FITNESS LANDSCAPES**

Nishant Gerald, Dibyendu Dutta, R. G. Brajesh, Supreet Saini*

Department of Chemical Engineering, Indian Institute of Technology Bombay

Powai, Mumbai – 400 076, India

* Corresponding author. Email: [saini@che.iitb.ac.in](mailto:saini@che.iitb.ac.in); Phone: 91 22 2576 7216


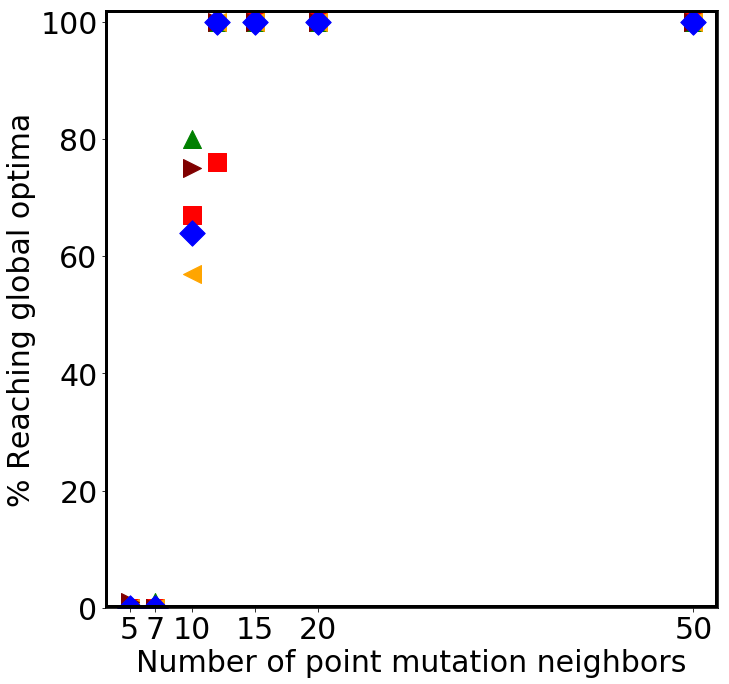

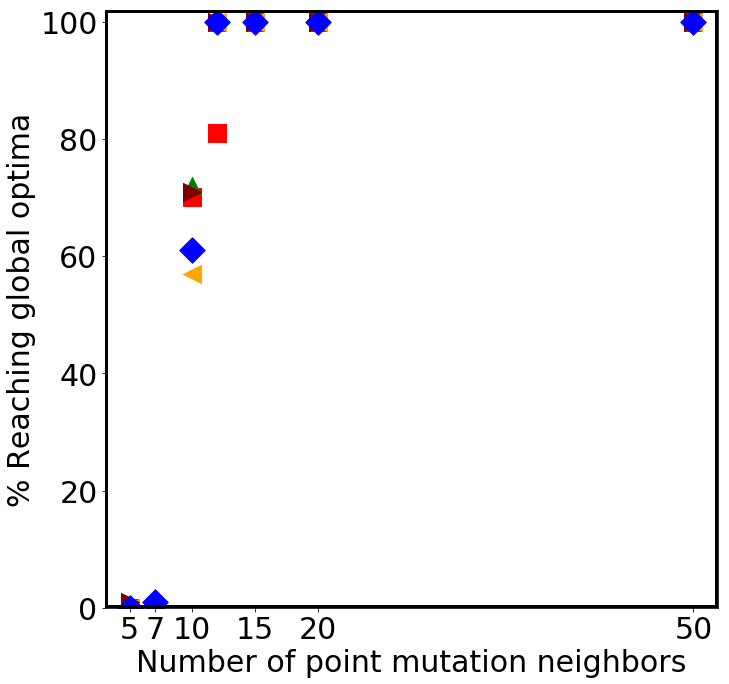

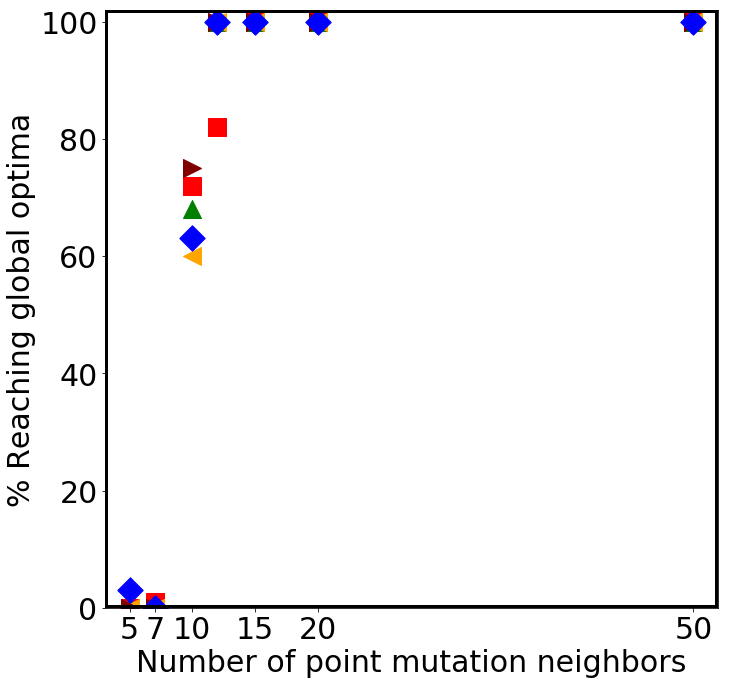

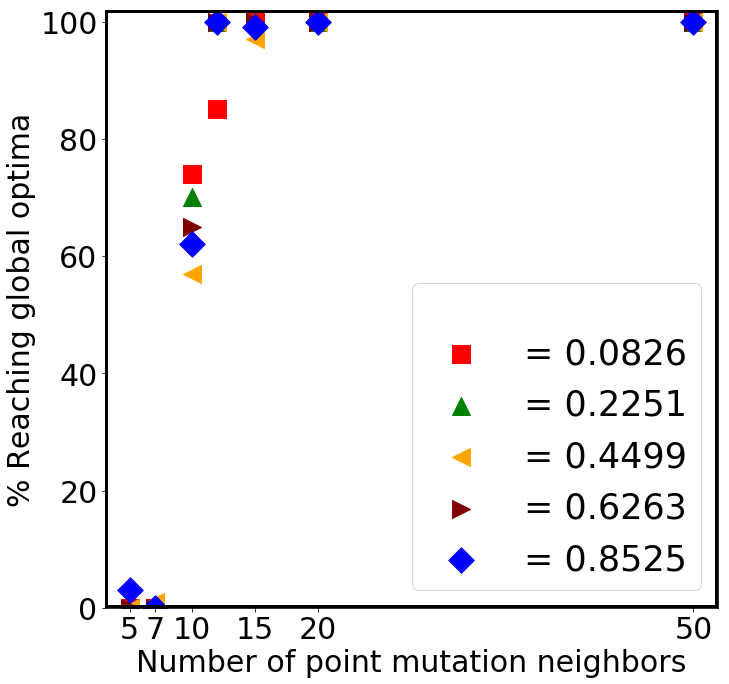


**(D)**

**(C)**

**(B)**

**(A)**

**Figure S1. The likelihood of reaching global optima increases from zero to one around critical value of K when the benefit is higher than the cost.** In this simulation the parameter **(A)** *a* is increased by a factor of 10; **(B)** parameter associated with cost per protein molecules (*α*) is increased by a factor of 10; **(C)** the parameter associated the sensitivity of the benefit function (*b*) is increased by a factor of 10; and **(D)** the parameter associated the sensitivity of the benefit function (*b*) is decreased by a factor of 10.

**Table S1. Parameter range of all 6 parameters (k, k_r_, k_d_, b, k_m_, bas) used in the simulations.**

| **Parameter** | **Lower bound** | **Upper bound** |
| --- | --- | --- |
| k | 12 | 60 |
| k_r_ | 0.1 | 0.3 |
| k_d_ | 0.001 | 0.005 |
| b | 0.1 | 4 |
| k_m_ | 9 | 100 |
| bas | 0.001 | 0.005 |
